# Enhanced plant disease classification with attention-based convolutional neural network using squeeze and excitation mechanism

**DOI:** 10.3389/frai.2025.1640549

**Published:** 2025-08-12

**Authors:** S. Karthikeyan, R. Charan, Sathiya Narayanan, L. Jani Anbarasi

**Affiliations:** ^1^School of Electronics Engineering, Vellore Institute of Technology, Chennai, India; ^2^School of Computer Science Engineering, Vellore Institute of Technology, Chennai, India

**Keywords:** edge computing devices, deep learning, convolutional neural network, plant disease classification, attention mechanism

## Abstract

**Introduction:**

Technology is becoming essential in agriculture, especially with the growth of smart devices and edge computing. These tools help boost productivity by automating tasks and allowing real-time analysis on devices with limited memory and resources. However, many current models struggle with accuracy, size, and speed particularly when handling multi-label classification problems.

**Methods:**

This paper proposes a Convolutional Neural Network with Squeeze and Excitation Enabled Identity Blocks (CNN-SEEIB), a hybrid CNN-based deep learning architecture for multi-label classification of plant diseases. CNN-SEEIB incorporates an attention mechanism in its identity blocks to leverage the visual attention that enhances the classification performance and computational efficiency. PlantVillage dataset containing 38 classes of diseased crop leaves alongside healthy leaves, totaling 54,305 images, is utilized for experimentation.

**Results:**

CNN-SEEIB achieved a classification accuracy of 99.79%, precision of 0.9970, recall of 0.9972, and an F1 score of 0.9971. In addition, the model attained an inference time of 64 milliseconds per image, making it suitable for real-time deployment. The performance of CNNSEEIB is benchmarked against the state-of-the-art deep learning architectures, and resource utilization metrics such as CPU/GPU usage and power consumption are also reported, highlighting the model’s efficiency.

**Discussion:**

The proposed architecture is also validated on a potato leaf disease dataset of 4,062 images from Central Punjab, Pakistan, achieving a 97.77% accuracy in classifying Healthy, Early Blight, and Late Blight classes.

## 1 Introduction

The importance of agriculture in everyday life cannot be minimized, as it offers an overabundance of direct and indirect benefits. Primarily, agriculture provides food for living beings as a direct benefit. Beyond this fundamental function, agriculture yields a range of indirect benefits that are equally vital. Moreover, agriculture significantly contributes to the Gross Domestic Product (GDP) showcasing its multifaceted impact on economy and the society (U.S. Department of Agriculture, 2020). Despite its positive contributions, there are challenges hindering the growth and production within this sector. One major issue is the occurrence of diseases in plants, which can impede growth and production. When a plant becomes diseased, not only does it suffer, but nearby plants are also at risk of infection, leading to a decline in overall production. Consequently, this affects both the GDP and the direct and indirect benefits associated with agriculture. Based on statistics, global crop loss is estimated at $220 billion (United States Dollars) annually, with plant disease accounting for 14.1% of this total (National Institute of Food and Agriculture, 2022). Biotic factors such as oomycetes, fungi, viruses, bacteria, nematodes, and viroids, along with abiotic elements like environmental factors contribute to this loss. Various rust diseases of wheat accounts to a revenue loss of approximately £60,000,000 worldwide, and India’s share is approximately £4,000,000 (Bayer Crop Science, n.d.). The Jowar crop affected by the Smut disease (SlideShare, n.d.), due to the fungus called Sphacelotheca Sorghi, resulted in a revenue loss of approximately £2,000,000 in the Mumbai region alone. Plant diseases affect crop quality, shelf life, and nutritional value, ultimately reducing yields and marketability. In addition, the impact of climate change and urbanization intensifies pressure on global food production systems. Earlier, manual techniques involving the identification of diseased areas and discerning the type of disease were carried out with the aid of experts and utilized a large amount of labour, time, and cost for the process. Manual disease classification is a slow and time-consuming process, often lacking accuracy, especially when dealing with numerous diseased plants. Several disease identification algorithms based on Artificial Intelligence (AI), Machine Learning (ML), and Deep Learning (DL) were introduced for automating the classification of diseased and healthy plants.

Automated plant disease classification has significantly improved the accuracy and efficiency of identifying crop issues. These advancements are helping farmers to make better decisions about plant health and management. With the integration of cutting-edge techniques, farming practices are made more sustainable, resilient, and resource-efficient. This paper proposes a deep learning architecture called Convolutional Neural Network with Squeeze and Excitation Enabled Identity Blocks (CNN-SEEIB), which integrates Squeeze-and-Excitation (SE) attention mechanisms within identity blocks for efficient plant disease classification. This light-weight model is ideal for edge devices with limited resources. It enhances feature learning by focusing on the most important information, resulting in improved classification accuracy. Designed for real-time inference, it ensures quick and efficient predictions.

The main contributions of the proposed CNN-SEEIB are as follows.

- The proposed CNN-SEEIB is a customized lightweight backbone with fewer parameters integrated with SE attention to enhance feature representation, enabling efficient, accurate, and real-time multi-label plant disease classification.
- A comparative study with 8 different pre-trained models demonstrated that CNN-SEEIB outperforms several state-of-the-art models. The accuracy of CNN-SEEIB was also compared with several state-of-the-art approaches for plant disease classification.
- The proposed model’s utilization of system resources, including CPU and GPU usage and power consumption, is evaluated and analysed as percentage metrics. The analysis of CNN-SEEIB in this study focuses on architecture optimization, which involves refining the traditional network structure and components to enhance overall performance.

With the growing demand for real-time and efficient solutions, the proposed CNN-SEEIB addresses the shortcomings of existing DL models, mainly in multi-label classification. The CNN-SEEIB architecture attained an optimized and efficient model for accurate leaf disease prediction. The proposed model resulted in better accuracy by leveraging visual attention to prioritize key features, without significantly increasing model complexity or size. Integrating SE blocks enhances feature representation and allows the model to adaptively highlight important features and suppress less significant ones, improving both accuracy and efficiency. The optimization of computational requirements, particularly for edge devices, is important for real-world agricultural applications where resource constraints are significant. Deploying the CNN-SEEIB model on portable edge devices such as drones or handheld diagnostic tools can enable real-time plant disease detection directly in the field.

The paper is structured as follows: Section 2 discusses the existing studies related to identifying and classifying plant diseases. Section 3 explains the proposed CNN-SEEIB model along with its computational complexity and highlights. Section 4 presents the results and discussion that details the dataset utilized, experimentation, and analysis of the obtained results. Section 5 concludes with the future scope.

## 2 Literature survey

Over the years, visual and image processing has seen a significant improvement in the number of algorithms in various domains, coinciding with the boom in the complexity of the problems addressed. In the realm of agriculture, for identifying and classifying plant diseases, a wide variety of techniques and methodologies have been proposed. With simple image processing and ML techniques, the field has progressed to embrace the power of AI and advanced DL approaches, offering comprehensive end-to-end solutions for addressing the challenges posed.

### 2.1 Image processing and machine learning based approaches

Vamsidhar et al. (2019) analysed co-occurrence feature computation and classifiers like K-means, Support Vector Machines (SVM), and MultiLayer Perceptron (MLP) for leaf disease classification. Leaf images were masked by filtering out green pixels, leaving behind the infected regions. Hybrid clustering, combining K-means and hierarchical clustering, segmenting the infected areas, further improved by a genetic algorithm for disease categorization. Co-occurrence features were selected for unsupervised ML techniques like K-means, while supervised methods like Naïve Bayes, SVM, and MLP were directly used without explicit feature selection, achieving an accuracy of 95.87% with SVM. Similarly, to identify disease in tomato plant leaves, Xian and Ngadiran (2021) proposed an Extreme Learning Machine (ELM) classifier with a single-layer feed-forward neural network. Harlick textures for feature extraction were used to the pre-processed image, and converted from Red-Green-Blue (RGB) to hue-saturation-value color space, resulting in an accuracy of 84.94%.

Singla et al. (2023) used machine learning approach using Neural Networks, SVM, and Naïve Bayes to classify plant leaves as healthy or infected. This method involved extracting features with a CNN, labelling the classes, and assigning probabilities through logistic regression. These classifiers were then used to determine the leaf categories, with the Neural Network classifier achieving an accuracy of 94% on the dataset. Ramesh et al. (2018) used a Random Forest (RF) classifier to distinguish between healthy and diseased papaya leaves. This method extracted feature descriptors using Histogram of Oriented Gradients, captured leaf edges with Hue moments, analysed texture using Haralick features, and represented pixel distribution with Color Histograms. The RF classifier trained on these features achieved an accuracy of 71%, outperforming other classifiers. Sabrol and Satish (2016) classified six categories of tomato leaves and stems, one healthy and five diseased by extracting shape, color, and texture features after image segmentation. Classification tree based on these features were developed, which achieved an overall testing accuracy of about 97.3%.

Thomkaew and Intakosum (2023) proposed a novel method for classifying plant leaves by combining CNN with the Scale Invariant Feature Transform (SIFT) algorithm. Leaf images were converted to binary followed by morphological transformations, like opening and closing that removed noise through erosion and dilation. Canny Edge detection was performed to identify edges in this processed images, and key-point features were extracted using SIFT. These processed images were then processed using a CNN for feature extraction, followed by classification using a Random Forest classifier. The approach achieved an accuracy of 95.62%.

### 2.2 Convolutional neural network based approaches

Deep learning techniques like CNNs has transformed computer vision, expanding its applications from classification to tasks like object detection, segmentation, and pose estimation, while increasing the algorithm performance across various fields (Alzubaidi et al., 2021). Specifically, CNNs have shown great success in identifying and classifying plant diseases (Tuğrul et al., 2022; Saleem et al., 2020; Maeda-Gutiérrez et al., 2020), leading to models that deliver impressive accuracy and efficient resource use. Batchuluun et al. (2022) introduced Periodic Implicit CNN (PI-CNN), a 30-layer CNN combining convolutional and residual blocks, which performed well on smaller datasets. They also developed PI-GAN, a Generative Adversarial Network (GAN) for augmenting dataset samples. Datasets like PlantVillage, PlantDoc, Fruits360, and Plants were evaluated based on the metrics like true positive rate, positive predictive value, F1-score, and accuracy.

Majji and Kumaravelan (2021) developed a CNN to classify 38 disease categories from the PlantVillage dataset, using only 16% of the total samples. The model, trained with a 90 to 10% split, batch size of 32, and 0.5 dropout over 200 epochs, attained an accuracy of 99.89%. Peyal et al. (2023) developed a lightweight 11-layer 2D-CNN for smartphone-assisted disease diagnosis, incorporated as an Android app called Plant Disease Classifier. This model classified 14 classes of tomato and cotton diseases with an average accuracy of 97.36% and an AUC of 99.9%. It also outperformed transfer learning models such as Inception V3, VGG16, VGG19, and MobileNet V1 and V2, while achieving a fast inference speed of 4.84 ms.

Imanulloh et al. (2023) designed a simple 12-layer CNN to classify plant diseases using the augmented PlantVillage dataset. Their model achieved accuracy, precision, recall, and F1-score of 97, 98, 97, and 97% respectively, outperforming other ImageNet models like VGG16, MobileNetV2, DenseNet121, ResNet50, and InceptionV3. Pandian et al. (2022) proposed a 14-layer deep CNN to classify 58 leaf disease classes across 16 plant species. They combined five datasets and applied augmentation techniques including Basic Image Manipulation (BIM), Neural Style Transfer (NST), and GANs to address class imbalance. Trained on multiple GPUs, this model achieved a classification accuracy of 99.97%, with weighted average precision, recall, and F1 scores of approximately 99.8%.

Naik et al. (2022) performed comparative analysis of twelve pre-trained deep learning architectures, such as VGG19 and DarkNet53, to classify five major chilli leaf diseases using a custom image dataset. VGG19 attained the highest accuracy of 83.54% on non-augmented data, whereas DarkNet53 outperformed others with an accuracy of 98.82% on augmented data. A custom squeeze-and-excitation-based CNN (SECNN) further improved performance, reaching 98.63% accuracy without augmentation and 99.12% with it. Furthermore, the SECNN model was evaluated on a plant leaf disease class from the PlantVillage dataset, resulting a robust generalization with an overall accuracy of 99.28%. Bhuyan et al. (2023) proposed SE_SPnet, a stacked parallel convolutional neural network integrated with a squeeze-and-excitation (SE) block for classifying rice leaf diseases. The architecture integrated multiple convolutional layers with varying kernel sizes to capture both local and global features, while the SE block enhanced relevant feature extraction. The model attained the accuracy. Sensitivity, specificity, precision, recall and F1-score as 99.2, 98.2, 98.5, 98.4, 98.2 and 98.5%, respectively, using SGD with momentum and 0.01 learning rate.

Naresh et al. (2024) proposed an optimized Squeeze-and-Excitation Densely Connected Convolutional Neural Network (SEDCNN) for early detection of chili leaf diseases. Among various SE block configurations, the integration of the standard SE block with SEDCNN achieved the best performance with 97% accuracy. With data augmentation, the model’s accuracy further improved to 98.86%, outperforming conventional CNNs, ResNet variants, and multiple transfer learning models. Chen et al. (2021) proposed SE-MobileNet, a hybrid model combining the lightweight MobileNet architecture with squeeze-and-excitation (SE) blocks to enhance plant disease identification. A two-phase transfer learning approach was used: the first phase involved training extended layers, followed by fine-tuning the entire model in the second phase. The SE-MobileNet model achieved a high accuracy of 99.78% on a public dataset with clear backgrounds and 99.33% under complex conditions with multiple disease classes.

Ashurov et al. (2025) proposed depthwise CNN model integrated with squeeze-and-excitation blocks, residual skip connections for accurate plant disease detection. The model attained an accuracy of 98% and an F1-score of 98.2%, resulting as a reliable tool for practical agricultural applications. Assaduzzaman et al. (2025) presented XSE-TomatoNet, an improved EfficientNetB0-based model enhanced with Squeeze-and-Excitation (SE) blocks and multi-scale feature fusion for precise tomato leaf disease classification. The model achieved 99.11% as accuracy and 99% precision and recall by 10-fold cross-validation.

Singh and Singh (2025) enhanced the ResNet50V2 model by integrating a Squeeze-and-Excitation (SE) block to improve accuracy in rice disease identification. Using the Kaggle rice leaf diseases dataset, the SE-enhanced model achieved a testing accuracy of 93.33%, outperforming the original ResNet50V2 which scored 83.61%. With a slight increase in parameters, the performance gain demonstrates the effectiveness of channel-wise attention through SE blocks.

The vine plant is economically important not only for grapes but also for products like wine, molasses, and culinary-use grape leaves. Diseases affecting grape leaves reduce yield and make the leaves unusable, leading to significant financial losses. Unal (2025) focused on classifying common grape leaf diseases such as scab and downy mildew alongside healthy leaves using deep learning models. The pre-trained networks performance was enhanced by integrating Convolutional Block Attention Module (CBAM) and Squeeze-and-Excitation (SE) blocks. These improvements increased the classification accuracy from 92.73 to 96.36%, demonstrating the effectiveness of attention-based enhancements. Nikhileswar et al. (2024) proposed custom CNN model named PlantLDNet optimized to distinguish diseased and healthy leaves while mitigating vanishing gradient problem. The model’s performance was compared with previous methods using metrics such as precision, recall, F1-score, ROC curves, and AUC. PlantLDNet attained F1-scores as 93% for Early Bright, 98% for Healthy, and 92% for Late Bright conditions.

### 2.3 Transfer learning based approaches

Optimizing pre-trained models (Pan and Yang, 2010) allows fine-tuning to fit specific tasks thus enhancing the performance through leveraging the strengths of the original models while saving training time (Balafas et al., 2023). Panchal et al. (2021) analysed transfer learning models like VGG16, ResNet50, and InceptionV3 for classifying leaf images into four infection stages (Healthy, Early, Middle, and End stages), using a PlantVillage dataset labelled with expert guidance. Among these, VGG16 achieved the highest accuracy of 93.5%. Parez et al. (2023) developed E-GreenNet, a customized version of MobileNetV3Small that enhanced its bottleneck layers. This model was trained and tested on PlantVillage, the Data Repository of Leaf Images (DRLI), and a combined PlantComposite dataset to classify leaves as healthy or infected. E-GreenNet outperformed other pre-trained models like VGG16, VGG19, MobileNetV1, and EfficientNetB0, achieving accuracies of 100% on PlantVillage, 96% on DRLI, and 99% on PlantComposite. Doğan (2023) trained MobileNetV3 on the PlantVillage dataset, which was augmented offline to 87,000 RGB images, attaining an accuracy of 99.85%, outperforming ResNet50, EfficientNetB3, and DenseNet121. Pramudhita et al. (2023) compared MobileNetV3Large and EfficientNetB0 on a strawberry leaf dataset with four classes (one healthy and three diseased). By tuning learning rate, epochs, and optimizers, they found that MobileNetV3Large, trained with a learning rate of 0.0001 for 70 epochs using the RMSProp optimizer, achieved the best accuracy of 92.14%.

### 2.4 Hybrid approaches and ensemble learning

Sahu et al. (2023) developed a hybrid deep learning and machine learning approach using Deep-Dream to classify tomato crops from the PlantVillage dataset. The Deep-Dream network was used to segment lesions in the images for better interpretability. These processed images were then analysed by 24 hybrid models, combining 8 feature extractors (EfficientNet B0-B8) with 3 machine learning classifiers (Random Forest, Stochastic Gradient Boosting, and AdaBoost). After hyperparameter tuning with Optuna, the best model used Deep-Dream with EfficientNet B4 as the feature extractor and AdaBoost as the classifier, achieving 96% accuracy on the dataset and 100% accuracy on a tomato leaf image database from the Indian Agricultural Research Institute. Fenu and Malloci (2023) applied ensemble learning to classify four classes of pear leaf diseases using a private field dataset called DiaMOS Plant (Ganaie et al., 2022). From four networks (EfficientNetB0, InceptionV3, MobileNetV2, and VGG19), the top three were selected to build ensemble models. Three pairs of two-network ensembles were created using bagging and weighted averaging. The best ensemble, combining EfficientNetB0 and InceptionV3, achieved the highest classification accuracy of 91.14%.

### 2.5 Light-weight architectures

Xu et al. (2022) proposed HLNet (High Speed and Light-weight Network), an optimized version of ShuffleNetV1 was designed to reduce computation time and FLoating-point OPerations (FLOPs). The model incorporated enhanced attention mechanisms, combining channel attention from the SE Network and spatial attention from the convolutional block attention mechanism, to limit computations and boost efficiency. Experiments were conducted on a private dataset combined with PlantVillage and the UC Irvine Machine Learning Repository, covering 28 leaf disease types across 20,490 images from six crop varieties. HLNet achieved 99.86% accuracy with an inference speed of 0.173 s. Thakur et al. (2023) analysed a lightweight Vision Transformer (ViT) (Han et al., 2023; Dosovitskiy et al., 2020) tailored for IoT-based agriculture applications. This model has fewer parameters than many state-of-the-art alternatives and achieved a testing accuracy of 98.86% and precision of 98.90%, outperforming existing models. Table 1 provides a summary of studies related to plant disease identification, highlighting that popular deep learning models like CNN offer various opportunities for enhancing both classification accuracy and execution speed.

**Table 1:** Summary of plant disease classification studies with methods, datasets, accuracy, technique type, and computational cost.

Ref. no.	Year of publication	Method	Dataset	Accuracy	Techniques (conventional ML or DL)	Computational cost (high/medium/low)
Vamsidhar et al. (2019)	2019	Co-occurrence features with SVM and MLP	Private dataset	95.87%	Conventional ML	Medium
Xian and Ngadiran (2021)	2021	Harlick textures and Feed-forward NN	Tomato disease from PlantVillage dataset	84.94%	DL	High
Ramesh et al. (2018)	2018	Histogram of Oriented Gradient with RF classifier	Private Dataset	71.00%	Conventional ML	Low
Sabrol and Satish (2016)	2016	Texture features	Tomato diseases	97.30%	Conventional ML	Medium
Thomkaew and Intakosum (2023)	2023	CNN and the SIFT algorithm	PlantVillage Dataset	95.62%	DL	Medium
Majji and Kumaravelan (2021)	2021	CNN	PlantVillage Dataset	99.89%	DL	Medium
Peyal et al. (2023)	2023	Lightweight 2D-CNN	PlantVillage Dataset	97.36%	DL	Medium
Imanulloh et al. (2023)	2023	12 layered CNN architecture	New Plant Diseases Dataset	97.00%	DL	High
Pandian et al. (2022)	2023	14-layered DCNN	Private Dataset	99.97%	DL	High
Panchal et al. (2021)	2021	Transfer learning with VGG1	PlantVillage dataset	93.50%	DL	High
Parez et al. (2023)	2023	CNN with MobileNetV3Small	PlantVillage, DRLI and a new Plant Composite (PC) dataset	100%	DL	High
Doğan (2023)	2023	MobileNetV3	PlantVillage Dataset	99.85%	DL	High
Pramudhita et al. (2023)	2023	MobileNetV3Large	Private Dataset	92.14%	DL	High
Sahu et al. (2023)	(2023)	Deep-Dream Network with EfficientNet B4	PlantVillage Dataset	96%	Mixed (DL and Conventional ML)	High
Fenu and Malloci (2023)	2023	Ensemble CNN with the EfficientNetB0 and InceptionV3	DiaMOS Plant dataset, a self-collected dataset	91.14%	DL	High
Xu et al. (2022)	2022	High Speed and Light-weight Network	Private Dataset	99.86%	DL	Medium
Thakur et al. (2023)	2023	Lightweight Vision Transformer	PlantVillage Dataset	98.86%	DL	Medium

## 3 Methodology

**Figure 1:**
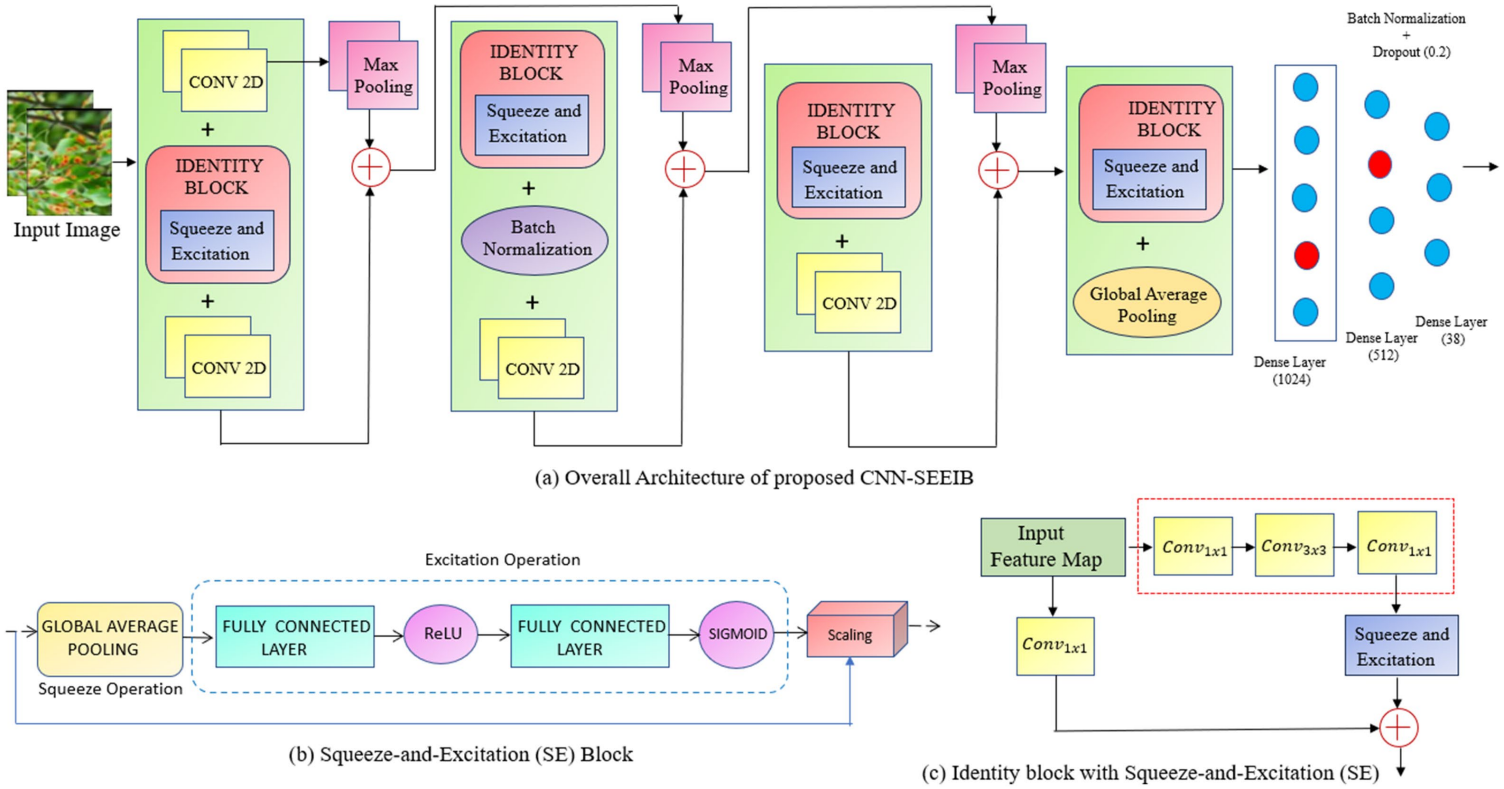
**(a)** The framework of the proposed CNN-SEEIB for plant disease classification. **(b)** Squeeze – and – Excitation (SE) Block. **(c)** Identity block with Squeeze – and – Excitation (SE).

The proposed methodology involves designing a customized architecture that integrates the Squeeze-and-Excitation (SE) attention mechanism with a deep neural network. Initially, a backbone model was constructed for classifying various plant disease classes, utilizing convolutional layers, residual/identity blocks, max-pooling, batch normalization, and dense layers. This identity-enhanced backbone is then combined with the attention mechanism to enhance performance and stability in multi-class classification. This approach facilitates accurate differentiation among multiple plant categories, including both healthy and diseased conditions. Figure 1 depicts the backbone framework employed for the multi-class classification of plant images.

### 3.1 CNN-SEEIB backbone architecture

The CNN-SEEIB backbone is a simple variant of the conventional CNN architecture, modelled to incorporate squeeze-and-excitation attention mechanisms. It consists of several fundamental components of CNNs: the input layer, convolutional blocks, identity blocks, max pooling, batch normalization, and activation layers.

#### 3.1.1 Input layer

The input layer accepts a three-channel image which is resized into patches of 224 × 224 × 3 pixels to standardize the input dimensions.

#### 3.1.2 Convolutional blocks

Each convolutional block performs 3 × 3 convolutions to extract local features from the input feature maps, followed by max pooling with a 2 × 2 window to reduce spatial dimensions and computational speed. This process enhances feature representation by transforming local features into more abstract global features as the network deepens. In CNN-SEEIB, there are four convolutional blocks that form the primary feature extractor.

#### 3.1.3 Identity blocks

The identity block is employed as a bottleneck design with a 1 × 1 convolution to reduce channel dimensions, then a 3 × 3 convolution processes these features, and finally another 1 × 1 convolution to restore the original channel size. The input of the block is then added element-wise to the output of the last convolution, facilitating the retention of essential features and avoid vanishing gradient issues through skip connections. This mechanism helps to maintain gradient flow and supports effective learning of complex features. CNN-SEEIB includes four such identity blocks interspersed with convolutional blocks.

#### 3.1.4 Max pooling layer

Max pooling, serves to down-sample input feature maps, enhancing computational efficiency and mitigating overfitting risks. It is typically applied post-convolutional layers to reduce spatial dimensions. During max pooling, each channel of the input feature map is processed independently using a pooling window, typically of size 2 × 2, where the maximum value within the region is extracted, discarding others. This process is iterated with a defined stride, dictating the window’s movement across the input feature map. Mathematically, the max pooling operation for a window of size x × x where stride of s can be expressed as given in Equation 1:


(1) $$\;\;\;\;M_{\mathrm{ijk}}=\max_{p=0}^{x-1}\;\max_{q=0}^{x-1}(N_{(\mathrm{si}+p),(\mathrm{sj}+q),k})\;$$

where $M_{\mathrm{ijk}}$ is the max pooling layer output at position (i,j) in channel k, and $N_{(\mathrm{si}+p),(\mathrm{sj}+q),k}$ refers the input value at position ( si+p,sj+q ) in channel k as stride s and x are the pooling window size.

#### 3.1.5 Batch normalization

Batch normalization normalizes the input activations across each mini-batch by subtracting the batch mean and dividing by the batch standard deviation, then applying learnable scaling and offset parameters. This normalization stabilizes and speeds up training while acting as a regularizer to reduce overfitting.

#### 3.1.6 Activation functions

The ReLU activation function, defined as max (0, x), introduces non-linearity after each convolution and fully connected layer, enabling the network to learn complex patterns. The final classification layer employs the SoftMax activation to output class probabilities.

#### 3.1.7 Global average pooling

Global Average Pooling (GAP) condenses the spatial dimensions of feature maps and delivers a concise feature representation. It includes computing the average of each feature map, yielding a single value for each channel, which can effectively reduce the network’s parameters and computational load. When applied to a feature map F of spatial position (i, j) with dimensions H×W×C (where H is height, W is width and C is the number of channels), the GAP operation generates a 1D output vector G with length C. The computation for the c th channel in the output vector G is given as Equation 2:


(2) $$G_{c}=\frac{1}{\mathrm{HXW}}\sum_{i=1}^{H}\sum_{j=1}^{W}F_{i,j,c}$$

This model used a global average pooling after the identity layer. GAP helps in making the network translation-invariant by summarizing the features in each channel, irrespective of their spatial location resulting in compact and informative feature representations.

Table 2 details the summary of the layers and parameters in the backbone CNN architecture. In CNN-SEEIB, the interaction between convolutional and identity blocks, enhanced by batch normalization and ReLU activation, ensures effective feature extraction and gradient flow. The model balances depth and computational efficiency with a total of approximately 3.34 million parameters, of which around 3.33 million are trainable, structured across 4 convolutional and 4 identity blocks.

**Table 2:** Layer-wise summary of the backbone CNN architecture with output shapes, specifications, and parameter count.

Layer type	Output shape	Details/specifications *Two sub-columns indicate two parallel processes which will be subjected to an add () before the next process		Total number of parameters
Input	224,224,3	Input image		0
Conv2D_Block_1	111,111,32	3×3 Conv2D, 32, Stride = 1, activation = relu 2×2 MaxPool, Stride = 1		896
Identity_Block_1	111,111,64	1×1 Conv2D, 32, Stride = 1, activation = relu + BatchNorm () 3×3 Conv2D, 32, Stride = 1, activation = relu + BatchNorm () 1×1 Conv2D, 64, Stride = 1, activation = None + BatchNorm ()	1×1 Conv2D, 64, Stride = 1, activation = None + BatchNorm ()	15,876
		Add () Activation (relu)		
Conv2D_Block_2	55,55,64	3×3 Conv2D, 64, Stride = 1, activation = relu 2×2 MaxPool, Stride = 1 ZeroPadding (1,0), (1,0)		36,928
Concatenate	55,55,96	Concatenate (Conv2D_Block_2, MaxPool (Conv2D_Block_1))		0
Identity_Block_2	55,55,128	1×1 Conv2D, 64, Stride = 1, activation = relu + BatchNorm () 3×3 Conv2D, 64, Stride = 1, activation = relu + BatchNorm () 1×1 Conv2D, 128, Stride = 1, activation = None + BatchNorm ()	1×1 Conv2D, 128, Stride = 1, activation = None + BatchNorm ()	67,592
		Add () Activation (relu)		
Batch Normalization				512
Conv2D_Block_3	27,27,128	3×3 Conv2D, 128, Stride = 1, activation = relu 2×2 MaxPool, Stride = 1 ZeroPadding (1,0), (1,0)		1,47,584
Concatenate	27,27,224	Concatenate (Conv2D_Block_3, MaxPool (Conv2D_Block_2))		0
Identity_Block_3	27,27,256	1×1 Conv2D, 128, Stride = 1, activation = relu + BatchNorm () 3×3 Conv2D, 128, Stride = 1, activation = relu + BatchNorm () 1×1 Conv2D, 256, Stride = 1, activation = None+BatchNorm ()	1×1 Conv2D, 256, Stride = 1, activation = None + BatchNorm ()	2,78,544
		Add () Activation (relu)		
Conv2D_Block_4	13,13,256	3×3 Conv2D, 256, Stride = 1, activation = relu 2×2 MaxPool, Stride = 1 ZeroPadding (1,0), (1,0)		5,90,080
Concatenate	13,13,480	Concatenate (Conv2D_Block_4, MaxPool (Conv2D_Block_3))		0
Identity_Block_4	13,13,512	1×1 Conv2D, 256, Stride = 1, activation = relu + BatchNorm () 3×3 Conv2D, 256, Stride = 1, activation = relu + BatchNorm () 1×1 Conv2D, 512, Stride = 1, activation = None + BatchNorm ()	1×1 Conv2D, 512, Stride = 1, activation = None + BatchNorm ()	11,30,528
		Add () Activation (relu)		
GlobalAveragePooling2D				0
Dense (1024), activation = relu				5,25,312
Dense (512), activation = relu				5,24,800
Batch normalization				2,048
Dropout (0.2)				0
Dense (38), activation = SoftMax				19,494

### 3.2 Improvisations through squeeze and excitation block

The utility of attention mechanisms enhances image recognition models by focusing on salient features within images, machine translation systems by highlighting relevant parts of a sentence. By enabling models to adapt their focus based on the input data, attention mechanisms improve the interpretability of neural network decisions and enhance the model’s reasoning process, boosting performance across diverse tasks (Hu et al., 2018; Woo et al., 2018). The SE is an attention mechanism used to adaptively reconfigure feature maps to enhance the representational power of CNNs. The SE block consists of two main functions: squeeze operation and excitation operation.

#### 3.2.1 Squeeze operation

To effectively capture inter-channel dependencies, the output features of each channel are analysed, as individual units within the transformation output L lack the capacity to extract global contextual features due to the constraints imposed by their limited receptive fields. Given a set of input feature maps $I_{f}\in R^{\text{HXWXC}}$ , where each ${I_{f}}^{c}\in R^{\mathrm{HXW}}$ , it becomes essential to squeeze spatial information across the entire feature map into a channel-wise representation. To address this, a global average pooling operation which is simple and effective is utilized to generate statistics for each channel. This operation compresses the spatial dimensions height H and width W of the input feature maps, resulting in a channel descriptor $s\in R^{c}$ Each component $s_{C}$ of this descriptor reflects the aggregated spatial response of the corresponding channel c and is derived as shown in Equation 3.


(3) $$s_{C}=F_{\text{Squeeze}}({I_{f}}^{c})=\frac{1}{H\;X\;W}\;\sum_{p=1}^{H}\sum_{q=1}^{W}{I_{f}}^{c}(p,q)$$

#### 3.2.2 Excitation operation

To enhance the model’s ability to adaptively recalibrate feature representation, the excitation phase is incorporated after the squeeze operation. The squeeze operation captures global spatial information by condensing each channel’s activation into a single scalar. The excitation phase utilizes this global context to effectively model channel inter-dependencies capturing the non-linear and non-mutually exclusive relationships between channels. To achieve this, a gating mechanism equipped with a sigmoid activation function is utilized. The excitation function is mathematically expressed in Equation 4:


(4) $$O=F_{\text{Excitation}}(s,T)=\sigma (\text{gating}(s,T))=\sigma (T_{2}\text{ReLU}(T_{1}s))$$

Here, $s\in R^{c}$ represents the channel-wise descriptors obtained from the squeeze operation, $T_{1}\in R^{\frac{c}{r}\times c}\;\text{and}\;T_{2}\in R^{c\times \frac{c}{r}}\;\;$ are the learnable weights of two fully connected (FC) layers, and r is the reduction ratio which controls the dimentionality compression and bottleneck capacity. The ReLU function introduces non-linearity, while the sigmoid function σ ensures, the output values (attention weights) lie within the range [0, 1].

This excitation mechanism consists of a lightweight two-layer MLP (multi-layer perceptron). The first FC layer reduces the channel dimensionality to $\frac{c}{r}$ , acting as a compression step, and is followed by a ReLU activation. The second FC layer restores the original channel dimension c, thereby resulting in the attention weights for recalibrating the original feature maps.

The recalibration is performed via channel-wise multiplication, where each channel of the transformation output is scaled by the corresponding excitation weight. This operation is described in Equation 5:


(5) $$\tilde{O_{\mathrm{Ic}}}=F_{\text{scale}}(O_{\mathrm{Ic}},z_{c})=z*O_{\mathrm{Ic}}$$

In this equation, $O_{\mathrm{Ic}}\in R^{H\;X\;W}$ denotes the feature map for channel c from the transformation output and $z_{c}\in [0,1]$ is the scalar excitation weight corresponding to that channel. The function $F_{\text{scale}}$ represents an element-wise channel scaling operation, which selectively emphasizes or suppresses channel responses based on their contextual importance.

As illustrated in Figure 1b, the complete Squeeze-and-Excitation (SE) Attention Block provides an efficient mechanism for dynamic channel-wise feature recalibration, enhancing representational capacity with minimal additional computational overhead. The proposed hybrid architecture integrates convolutional blocks with identity blocks (Figure 1c), enabling deeper models while mitigating the vanishing gradient problem through skip connections. Concatenation with max-pooling operations facilitates multi-scale feature representation across different levels of abstraction, thereby enhancing the model’s capability to extract rich and discriminative features. Batch normalization is utilized to stabilize and accelerate training by normalizing intermediate features thus improving convergence and generalization. To reduce overfitting and computational complexity, global average pooling is incorporated prior to the dense layers for effectively decreasing the number of parameters.

The integration of the Squeeze-and-Excitation mechanism enhances the model’s representational power by modelling channel-wise interdependencies. The *squeeze* operation utilizes global average pooling to capture spatially global information, while the *excitation* operation computes adaptive channel weights that are used to recalibrate feature maps. This selective importance on informative channels allows the network to focus on the most task-relevant features, effectively suppressing redundant or noisy information. By embedding the SE block, the model achieves superior feature discrimination, leading to improved classification accuracy and robustness. This attention-driven enhancement makes the model more resilient to variations in input data, which is evident from the improved experimental metrics compared to baseline architectures.

### 3.3 Computational complexity

In general, the overall runtime of the convolution blocks in CNN is linear, and therefore for classifying images with n pixels, the computational complexity of the feature extractor blocks is O(n) . This is due to the fact that the complexity of the convolution operation is O(n) and the other operations like pooling and SE do not take more than the linear time. As given in Lux (n.d.), if the extracted feature map is of dimension N , the dense neural network layer (i.e., feed forward neural network) has a computational complexity of $O(N^{4})$ . Therefore, the complexity of the proposed CNN-SEEIB is $O(N^{4}+n)\approx O(N^{4})$ since $n\ll N^{4}.$ Note that the computational complexity of the proposed architecture is comparable to that of the feed forward neural network.

## 4 Results and discussion

This section details (i) Dataset Description, which outlines the dataset’s characteristics, (ii) Experimentation, detailing the experimental setup, training parameters, and evaluation criteria, and (iii) Result Analysis, which interprets the obtained outcomes, including training and validation results, testing outcomes, inferencing outcomes, and comparisons with other models.

### 4.1 Dataset description and processing

**Figure 2:**
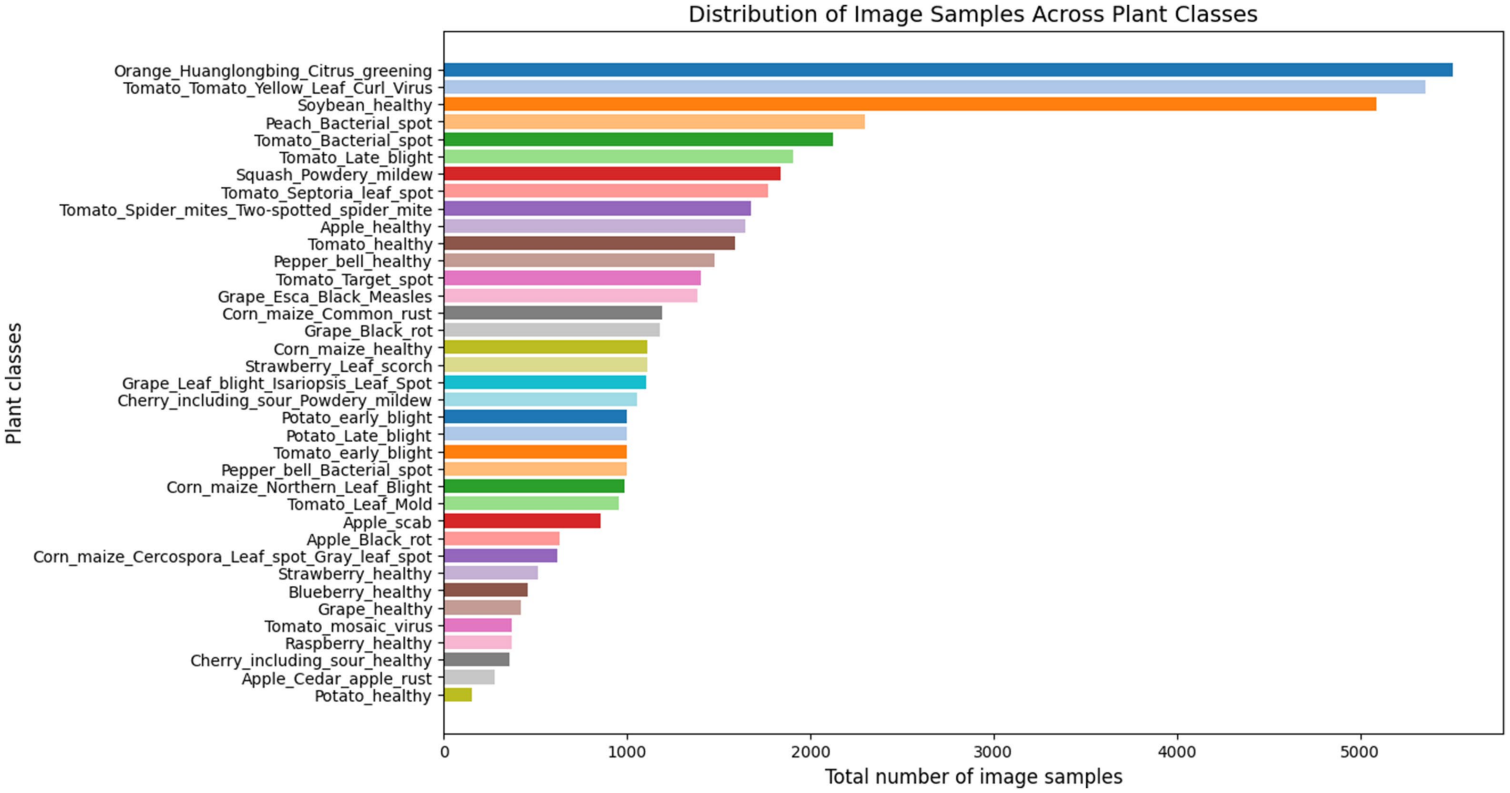
Class wise distribution of the PlantVillage dataset.

**Figure 3:**
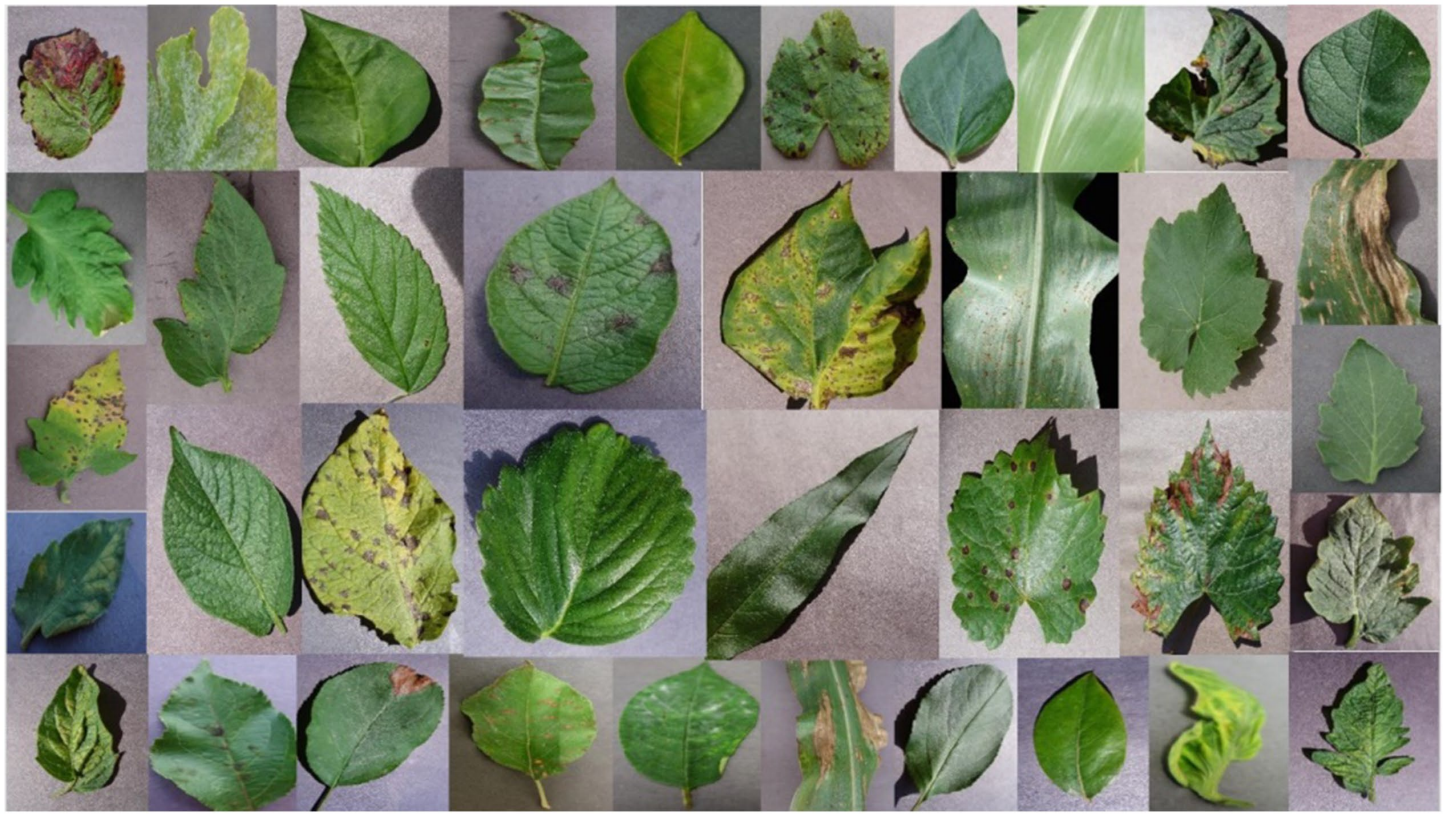
Sample leaf images from each of the 38 classes from the PlantVillage dataset.

This proposed work analysed the widely used PlantVillage dataset, renowned for its extensive and diverse collection of plant leaf images (Mohanty et al., 2016). The dataset contains a total of 54,305 images representing 38 distinct classes, which include 14 crop species such as apple, blueberry, cherry, grape, orange, peach, pepper, potato, raspberry, soy, squash, strawberry, and tomato. These 38 classes are categorized into 17 fungal diseases, 4 bacterial diseases, 2 diseases caused by mold (oomycetes), 2 viral diseases, 1 disease caused by a mite, and 12 classes representing healthy crops. Figures 2, 3 illustrate the distribution of classes across the dataset and shows the representative leaf samples from each of the 38 categories. Prior to training, the dataset undergoes a preprocessing process involving the removal of duplicate entries and validation of file formats. All images are then resized to 224 × 224 × 3 and the dataset is split into training (80%), validation (10%), and testing (10%) subsets to support model training and evaluation.

### 4.2 Experimental setup

The training, testing, and inference experiments were conducted using Google Collaboratory with a T4 cloud GPU backend. The implementation was carried out in Python 3.10, utilizing TensorFlow-Keras 2.15.0 as the deep learning framework. Model inference was performed on a local machine equipped with an Intel (R) Core (TM) i7 CPU, 16GB RAM, and an NVIDIA GeForce GTX 1650 Ti GPU with 4GB of dedicated memory. To monitor hardware performance metrics such as CPU and GPU utilization and power consumption, HWiNFO software was employed. The software tracks four key parameters: total CPU usage, GPU D3D usage, CPU package power, and GPU power. For evaluation, 100 samples of each parameter were collected over a 3-min interval, and the average values were calculated. Additionally, the net_flops (model) function from the net_flops Keras library was used to compute the FLOPS (floating point operations per second) of the model.

### 4.3 Hyperparameter tuning

The CNN-SEEIB model was fine-tuned to improve its performance by adjusting several key hyperparameters, like dropout rate, batch size, epochs, learning rate, and gradient optimizer. This study employed the Adam optimizer (adaptive moment estimation) (Kingma and Ba, 2015), which is known for its efficiency and low memory requirements. Adam combines the benefits of gradient descent with momentum and the RMSProp optimizer (GeeksforGeeks, n.d.), enhances the plant leaf classification. Table 3 details the list of hyperparameters that are tuned to attain better results.

**Table 3:** Hyperparameters for the proposed CNN-SEEIB model.

Hyperparameter	Option/value
Optimizer	Adam
Epochs	50
Batch size	32
Learning rate	0.001
Learning rate decay	0.5

The loss during Back Propagation is computed using Categorical Cross entropy loss function as defined as Equation 6.


(6) $$L(y,\hat{y})=\frac{1}{N}\sum_{i=1}^{N}\sum_{k=1}^{K}y_{i,k}\log({\hat{y}}_{i,k})$$

where y is the ground truth distribution (one-hot encoded labels), $\hat{y}$ is the predicted probability distribution (the output of the SoftMax activation function), $y_{i,k}$ is the true label for the ith sample in class k, and ${\hat{y}}_{i,k}$ is the predicted probability for the ith sample belonging to class k.

### 4.4 Evaluation metrics

Evaluation metrics play a pivotal role in assessing the efficacy of the developed and trained model. In this study, the evaluation metrics are categorized into two groups: (i) testing metrics and (ii) inferencing metrics.

#### 4.4.1 Metrices for testing

The classification performance of the proposed model was evaluated based on four key metrics derived from the confusion matrix: accuracy, precision, recall and F1-score. While accuracy indicates the overall percentage of correct predictions, there might be instances where false predictions have different implications. In such scenarios, precision, recall and F1-score provide a more comprehensive evaluation of the model.

True Positives (TP) refer to the number of instances where the model correctly identifies diseased leaves as belonging to a disease class, i.e., actual positives correctly predicted as positives. In the context of the PlantVillage dataset, this means the model accurately detects the presence of a specific leaf disease. True Negatives (TN) denote the number of healthy leaf samples that are correctly identified as healthy, i.e., actual negatives predicted as negatives. False Positives (FP) represent healthy leaves that are incorrectly classified as diseased, i.e., actual negatives mistakenly predicted as positives. False Negatives (FN) indicate diseased leaves that the model incorrectly classifies as healthy, i.e., actual positives predicted as negatives.

The definitions of the evaluation metrics are given in Equations 7–10:


(7) $$\text{Accuracy}=\frac{\mathrm{TP}+\mathrm{TN}}{\mathrm{TP}+\mathrm{TN}+\mathrm{FP}+\mathrm{FN}}\times 100$$

(8) $$\text{Precision}\;(P)=\frac{\mathrm{TP}}{\mathrm{TP}+\mathrm{FP}}$$

(9) $$\text{Recall}\;(R)=\frac{\mathrm{TP}}{\mathrm{TP}+\mathrm{FN}}$$

(10) $$F1\;\text{Score}=\frac{2\times P\times R}{P+R}$$

#### 4.4.2 Metrics for inferencing

To assess the computational capabilities, the following inference metrics were considered: (i) number of parameters and model size, (ii) inference time and (iii) frames per second.

##### 4.4.2.1 Number of parameters and model size

These metrics depend upon the architecture of the model based on the number of trainable and non-trainable parameters, and the memory occupied by these parameters during when the model is loaded.

#### 4.4.3 Inference time

It refers to the duration of time required for a model to process single input and generate predictions. It measures the computational efficiency of the model.

#### 4.4.4 Frames per second

Frames Per Second (FPS) measures the number of frames a model can process and render per second. A higher FPS indicates smoother performance and better visual quality and is a critical factor in real-time applications.

### 4.5 Performance evaluation

During the training phase, the proposed CNN-SEEIB architecture, along with eight different pre-trained models, was trained on the dataset for 50 epochs using the same set of hyperparameters. The pre-trained models used include VGG16, ResNet50, EfficientNetB0, DenseNet121, MobileNetV2, InceptionV3, Inception-ResNetV2, and XceptionNet. Table 4 presents the accuracy of each model along with the input dimensions for both training and validation.

**Table 4:** Training and validation accuracy comparison of the proposed CNN-SEEIB model and pre-trained models.

Model	Input shape	Training set accuracy	Validation set accuracy
VGG16	(224,224,3)	100.0%	95.29%
Resnet50	(224,224,3)	100.0%	99.65%
Efficient NetB0	(224,224,3)	100.0%	99.87%
DenseNet121	(224,224,3)	100.0%	99.83%
MobileNetV2	(224,224,3)	100.0%	99.78%
InceptionV3	(299,299,3)	100.0%	99.89%
Inception-ResnetV2	(224,224,3)	100.0%	99.83%
Xception	(224,224,3)	100.0%	99.83%
Proposed CNN-SEEIB	(224,224,3)	100.0%	99.89%

**Figure 4:**
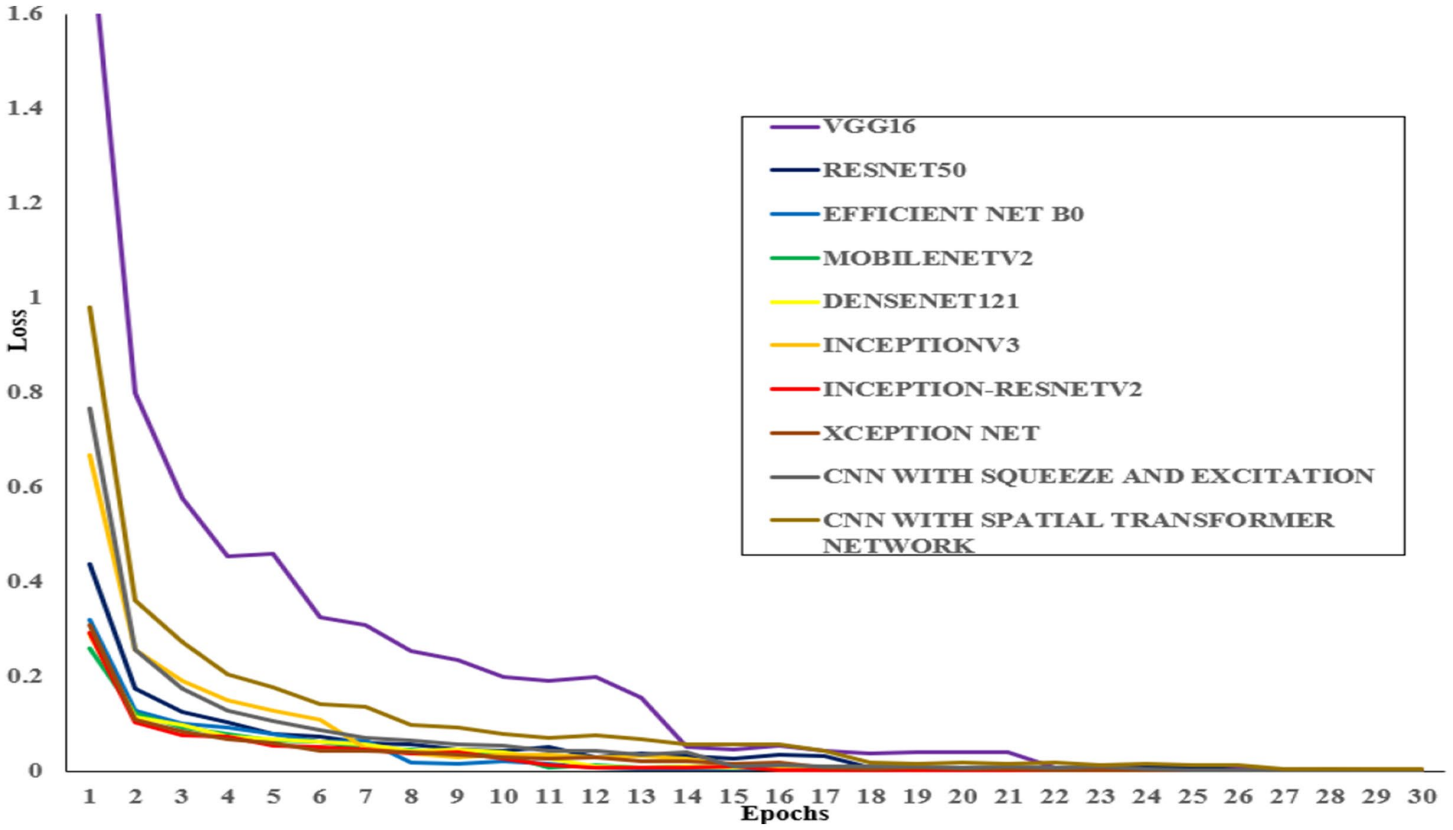
Training loss curves for pre-trained models and the proposed CNN-SEEIB model.

**Figure 5:**
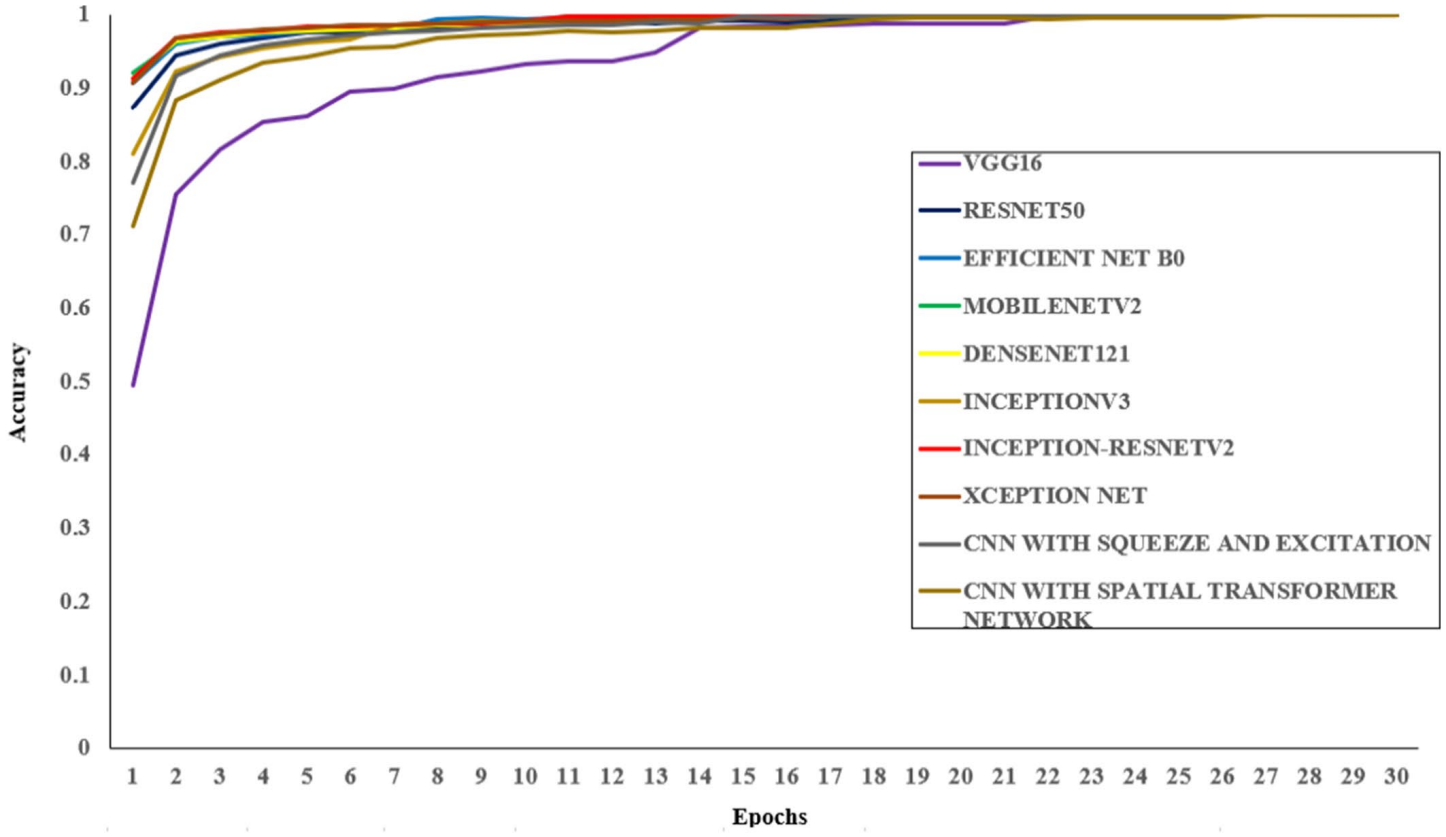
Training accuracy curves for pre-trained models and the proposed CNN-SEEIB model.

All models were trained to achieve optimal accuracy on the training dataset, incorporating regularization techniques to prevent overfitting. The selection of models represents a range of architectural improvements ranging from depth-scaling design of VGG16 to the more intricate scaling strategies employed by EfficientNet-B0. ResNet-50 and DenseNet-121 were chosen for their robust deep architectures leveraging residual and dense connections, respectively. MobileNet-V2 provides a low-weight solution that is appropriate for data with limited constraints, making it perfect for applications that require real-time processing. Inception-V3 and Inception-ResNetV2 utilise multi-scale processing and hybrid architectures to successfully capture a wide range of features. These models encompass early stopping, to halt training upon validation accuracy saturation, and model checkpoints to save the model weights with the highest accuracy in the validation set. As shown in Table 4, the proposed CNN-SEEIB model achieved the highest validation accuracy among all compared models, whereas VGG16 recorded the lowest. In addition to the enhanced proposed model, only InceptionV3 reached a peak accuracy of 99.89%. Figures 4, 5 illustrate the training loss and accuracy curves for each pre-trained model as well as the CNN-SEEIB model.

Table 5 presents the comparative results of multi-class classification performance on the test dataset between the proposed CNN-SEEIB model and the pre-trained models. The CNN-SEEIB model outperformed all others, achieving an accuracy of 99.79%, with precision, recall, and F1-score values of 0.9972, 0.9970, and 0.9971, respectively. The incorporation of the SE attention mechanism enhanced the model’s ability to focus on critical features. Among the pre-trained models, DenseNet121 demonstrated the best performance across all evaluation metrics, while VGG16 exhibited the lowest.

**Table 5:** Performance comparison of models based on accuracy, precision, recall, and F1-score.

Model	Accuracy	Precision	Recall	F1-score
VGG16	94.94%	0.9428	0.9256	0.9331
Resnet-50	99.45%	0.9931	0.9919	0.9924
Efficient Net-B0	99.69%	0.9952	0.9950	0.9951
DenseNet-121	99.76%	0.9960	0.9969	0.9965
MobileNet-V2	99.62%	0.9946	0.9948	0.9947
Inception-V3	99.73%	0.9960	0.9959	0.9962
Incpetion-ResnetV2	99.74%	0.9959	0.9959	0.9960
Xception	99.70%	0.9949	0.9957	0.9953
Proposed CNN-SEEIB	99.79%	0.9970	0.9972	0.9971

Models that deliver high classification performance while maintaining low resource consumption are considered optimal. Through inferencing, the resource utilization metrics such as model size, inference time, and frames per second are measured and are evaluated. Table 6 show the results of this comparison based on inference performance. The measurement of the metrics has been carried out in a local runtime to avoid latency errors in measurement. The model size and parameters are obtained through model statistics when the model is loaded while the inference time and frames per second metrics are measured as average over time when the model is evaluated in real-time. It is clear that mobilenetV2 has the lowest inference time, Inception-ResnetV2 has the highest number of parameters and memory, and DenseNet121 takes the largest inference time. The proposed model improvised through SE mechanism stands next to MobileNetV2 in inference time and differs only by a small margin. The proposed CNN-SEEIB has the smallest size and number of parameters and performs faster than most of the pre-trained models except for MobileNetV2.

**Table 6:** Inference performance and resource utilization comparison of models.

Model	Number of parameters/model size (MB)	Inference time (ms)	Frames per second (FPS)	Floating point operations per second (FLOPS)
VGG16	15,668,070/59.77 MB	68	14.5	15.347
Resnet-50	25,724,838/98.13 MB	72	13.5	3.864
Efficient Net-B0	4,098,249/15.63 MB	69	14.5	0.385
DenseNet-121	7,076,454/26.99 MB	80	12.3	2.839
MobileNet-V2	3,267,814/12.47 MB	62	16.5	0.567
Inception-V3	23,939,910/91.32 MB	75	12.5	5.784
Inception-ResnetV2	54,395,142/207.50 MB	70	13.5	6.551
Xception	20,939,342/79.88 MB	71	13.5	3.601
Proposed CNN-SEEIB	3,340,194/12.74 MB	64	16.5	2.121

**Figure 6:**
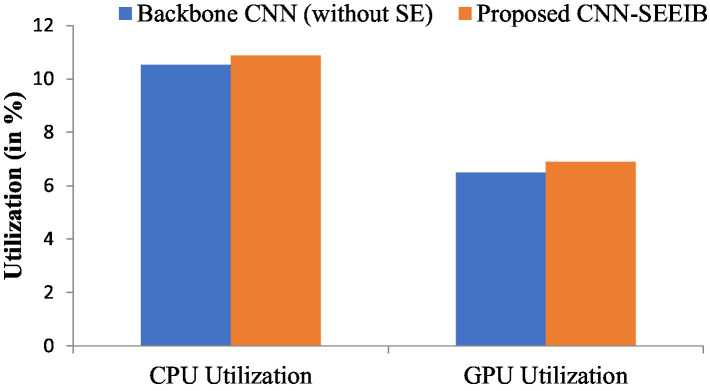
CPU and GPU utilization between the backbone CNN (without SE) and the proposed CNN-SEEIB.

**Figure 7:**
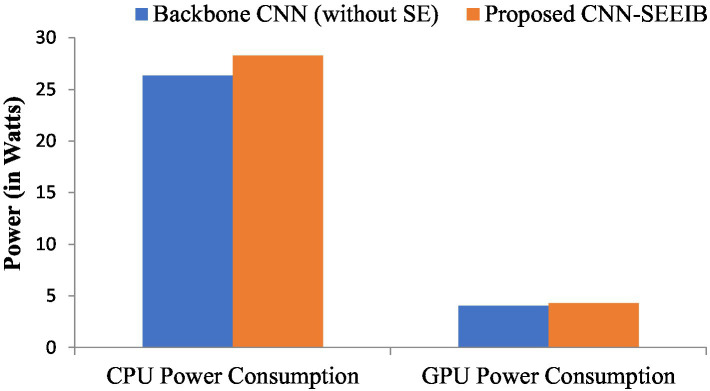
CPU and GPU power consumption between the backbone CNN (without SE) and the proposed CNN-SEEIB.

Resource utilization and power consumption metrics are vital for assessing the feasibility of deploying the CNN-SEEIB model on edge devices (Chen and Ran, 2019). To evaluate system resource usage, CPU and GPU utilization percentages serve as key metrics, while the power consumption of both CPU and GPU (measured in watts) indicates the load the model imposes on the hardware. Although these metrics do not directly impact the classification task, they influence the hardware or system where the model operates. Figures 6, 7 illustrate the resource utilization details for the proposed CNN-SEEIB and its backbone CNN architecture. The results show that integrating the SE mechanism in CNN-SEEIB leads to a 0.35% increase in CPU utilization and a 0.4% increase in GPU utilization. Similarly, CPU power consumption rises by approximately 7%, and GPU power consumption by about 6%. However, these increases are justified by the notable gains in classification accuracy achieved by the model. Moreover, the efficient design of CNN-SEEIB keeps overall GPU utilization relatively low by optimizing resource use and minimizing computations.

In resource-constrained environments, these modest trade-offs are reasonable, given the enhanced decision-making accuracy of the model. Potential further optimizations, such as model quantization or pruning, could reduce resource consumption without sacrificing performance, improving its suitability for edge deployment. Additionally, as energy-efficient hardware becomes more accessible, the practical impact of such increases will diminish. These considerations underscore the balance between accuracy and efficiency, making CNN-SEEIB a scalable and sustainable solution for various real-world applications.

### 4.6 Visualization of activation maps

**Figure 8:**
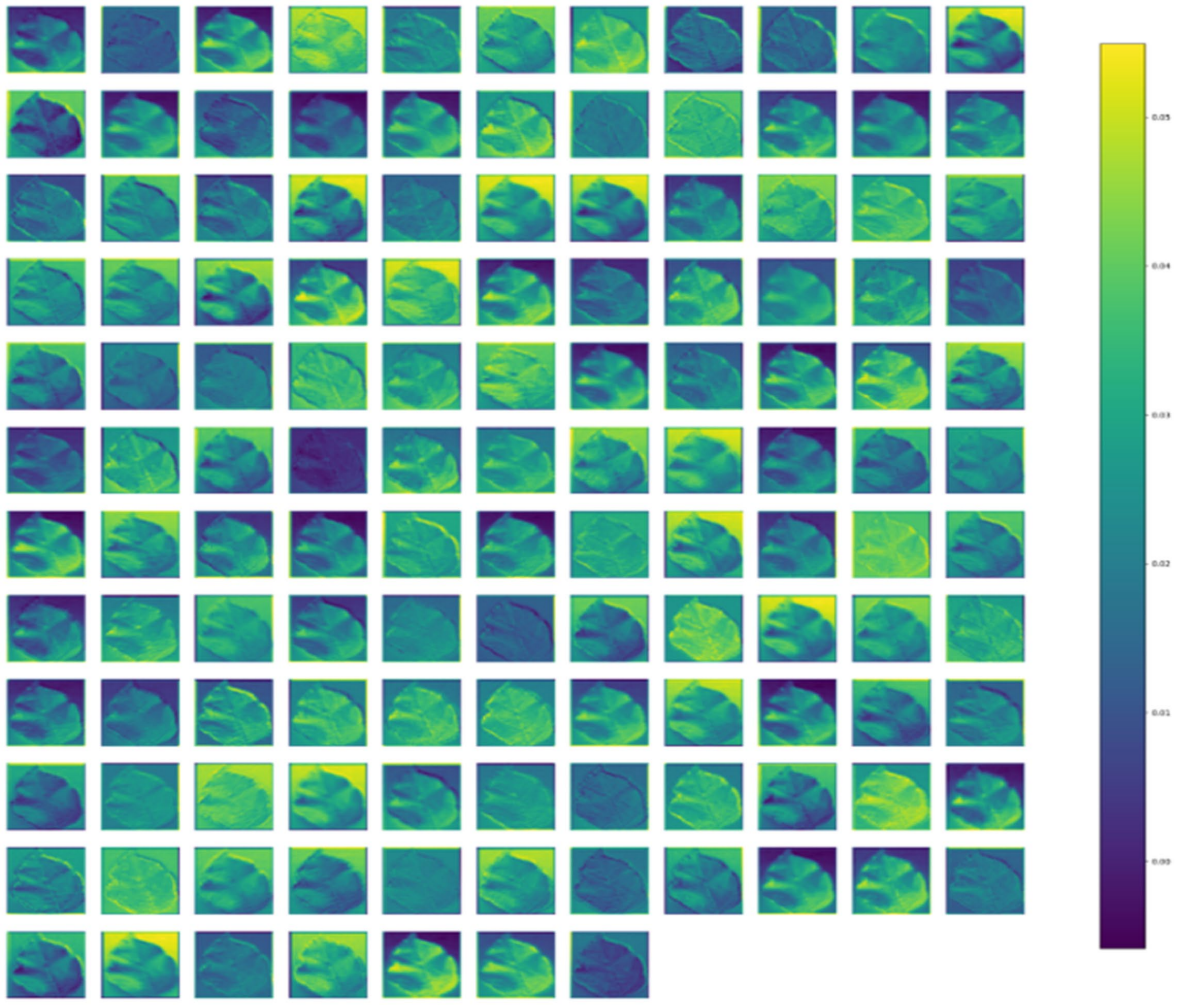
Intermediate feature maps obtained from the CNN-SEEIB model.

Activation Maps offer valuable insights into the features captured by the model internally, shedding light on its function and behavior. As features flow from the input to the output layer, they become increasingly global and abstract, a process comprehended by the dense layers for accurate classification. Figure 8 visually represents the intermediate feature maps of the proposed CNN-SEEIB model, illustrating the progression of features through the network.

### 4.7 Ablation study

**Figure 9:**
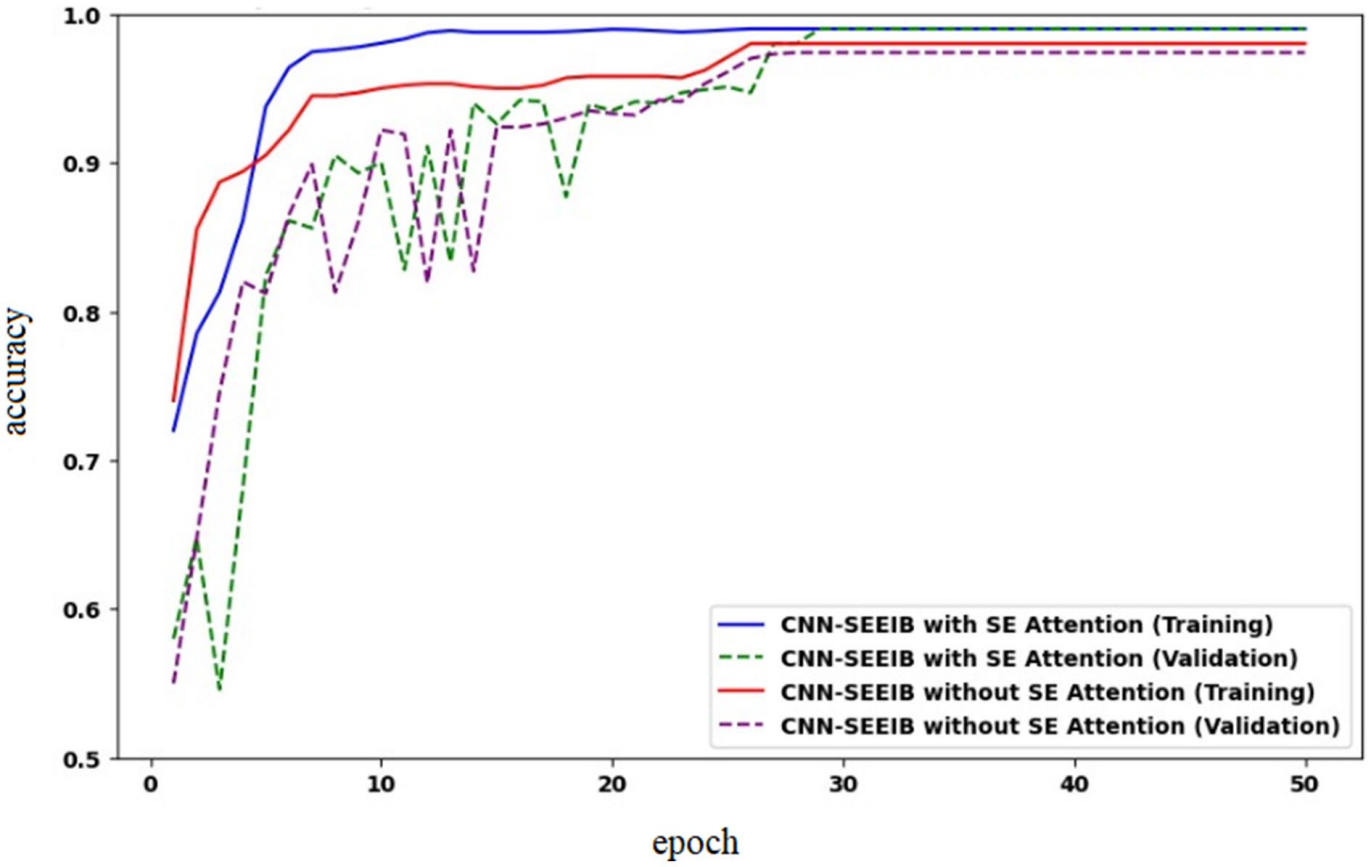
Model accuracy of CNN-SEEIB with and without squeeze and excitation (SE) attention.

To analyze the impact of the SE mechanism on the proposed CNN-SEEIB model, an ablation study was conducted by assessing the model’s performance without the SE module. Figure 9 presents the accuracy curves for CNN-SEEIB both with and without SE during training and validation. It is observed that, without SE, the validation accuracy lags behind the training accuracy even at the 50th epoch. In contrast, with the SE mechanism included, the validation accuracy aligns closely with the training accuracy from the 35th epoch onward. This demonstrates that the SE module significantly contributes to the accuracy improvement of the proposed CNN-SEEIB model.

### 4.8 Performance comparison with the state-of-the-art

The proposed model in the study is further compared with several other architectures from existing studies to highlight its significance and performance as shown in Table 7.

**Table 7:** Comparison of accuracy results with the existing state-of-the-art models.

Ref. no	Year of publication	DL model used	Accuracy	F1-score
Ahmad et al. (2021)	2021	MobileNetV3-large	99.69%	0.9962
Yao et al. (2024)	2024	Generalized Stacking Multi-output CNN (with InceptionV3)	99.56%	0.9956
Guo (2023)	2023	MobileNetV2	99.41%	0.9941
Vo et al. (2023)	2023	Ensemble of EfficientB0 and MobileNetV2	99.77%	0.9979
Hassan et al. (2021)	2021	EfficientNetB0	99.56%	0.9961
Proposed work		CNN-SEEIB	99.79%	0.9971

Each model achieved peak accuracies ranging from 99.41 to 99.79%, demonstrating their effectiveness in accurately identifying and classifying plant diseases. The proposed CNN-SEEIB, outperformed all others, reaching the highest accuracy of 99.79%. The model proposed in these studies (Hassan et al., 2021; Brahimi et al., 2018) diverges from traditional transfer learning approaches as it leverages the custom hybrid architecture incorporated with the SE mechanism that significantly enhances the model’s capability to discern intricate features and patterns within plant images. Despite the sophisticated design, the model remains lightweight, offering a remarkable balance between performance and computational efficiency. The high accuracy attained highlights the model’s potential for practical deployment in agricultural settings, where accurate and efficient disease identification is crucial for ensuring crop health and yield.

### 4.9 Validation of robustness and generalizability

Despite the homogeneous background and controlled conditions of the PlantVillage dataset, it has been widely utilized in numerous studies as a benchmark for plant disease classification. The class imbalance issue with this dataset is a potential challenge as certain disease classes contain more samples than others, potentially resulting in biased predictions. The datasets may not fully reflect actual agricultural contexts because they were collected in lab settings with uniform backgrounds and ideal illumination. Furthermore, the dataset has noise associated with the labels, which allows DL models to alter prediction biases rather than learning the disease features itself. This could potentially impact the performance of the models in real-world scenarios. To validate the robustness and the generalization of the proposed CNN-SEEIB model, the experiment is repeated on the potato leaf disease dataset (Rashid et al., 2021). Early detection of potato leaf diseases is challenging due to the diversity in crop species, disease symptoms, and varying climatic conditions, which add complexity to the detection process (Rashid et al., 2021). The potato leaf disease dataset comprises of 4,062 images collected from the Central Punjab region of Pakistan.

**Figure 10:**
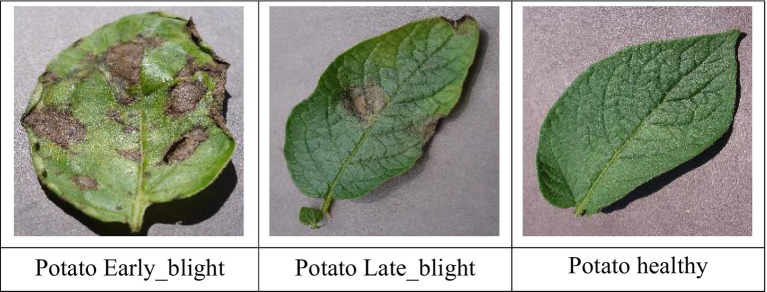
Sample potato leaf disease images.

This dataset comprises a diverse collection of images categorized into three distinct classes: early blight, late blight, and healthy each representing a specific condition affecting potato crops. Early blight, caused by the fungus *Alternaria solani*, typically affects older plants and is also known as target spot. Late blight, caused by *Phytophthora infestans*, is a severe disease that can lead to rapid crop failure without proper control measures. In contrast, healthy potatoes are nutrient-rich, providing essential elements like vitamin C and potassium. The dataset was divided into 3,251 training images, 416 testing images, and 405 validation images. The sample potato leaf images used for training and validation is shown in Figure 10.

**Figure 11:**
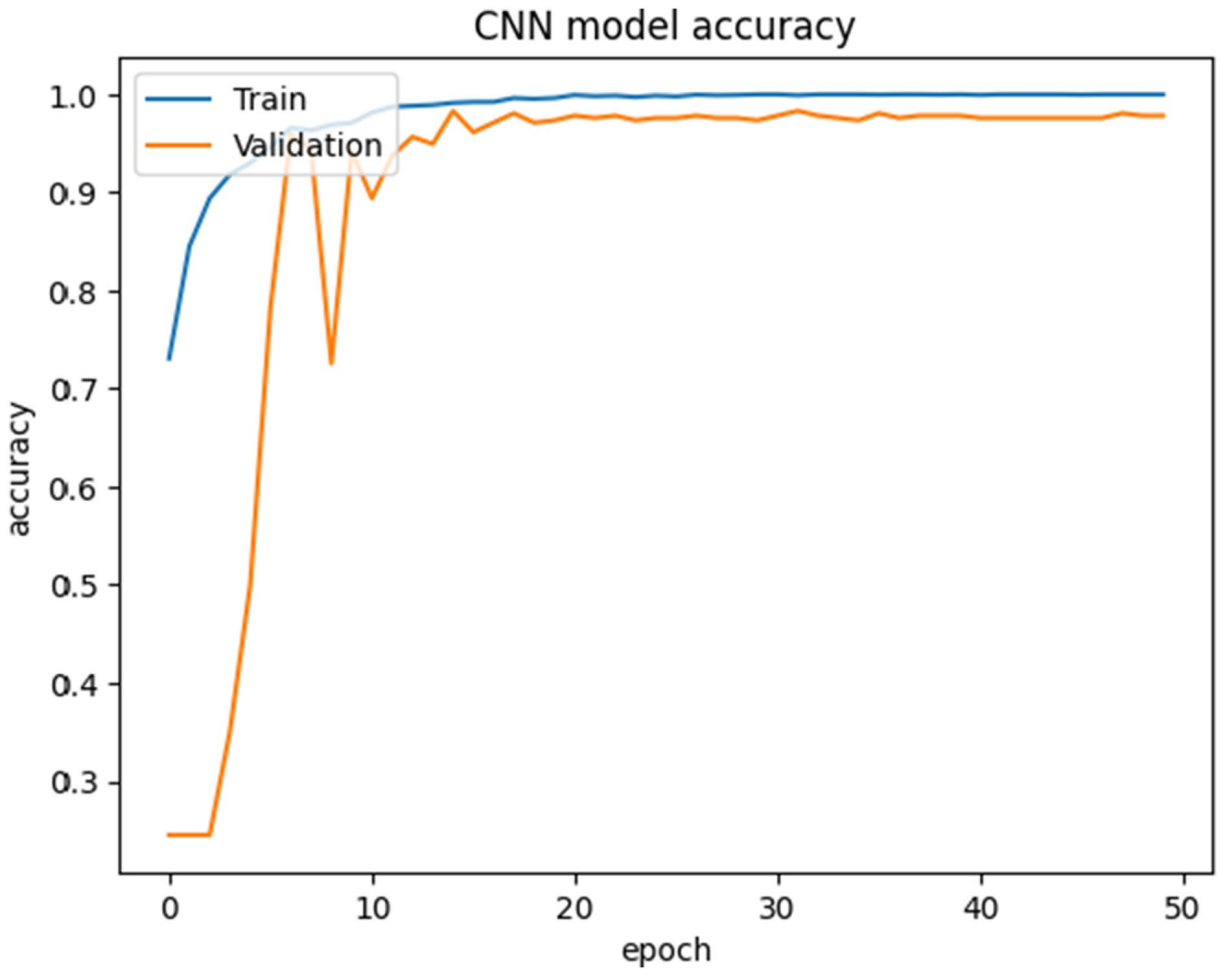
Accuracy plot for CNN-SEEIB’s performance on potato leaf disease dataset.

**Figure 12:**
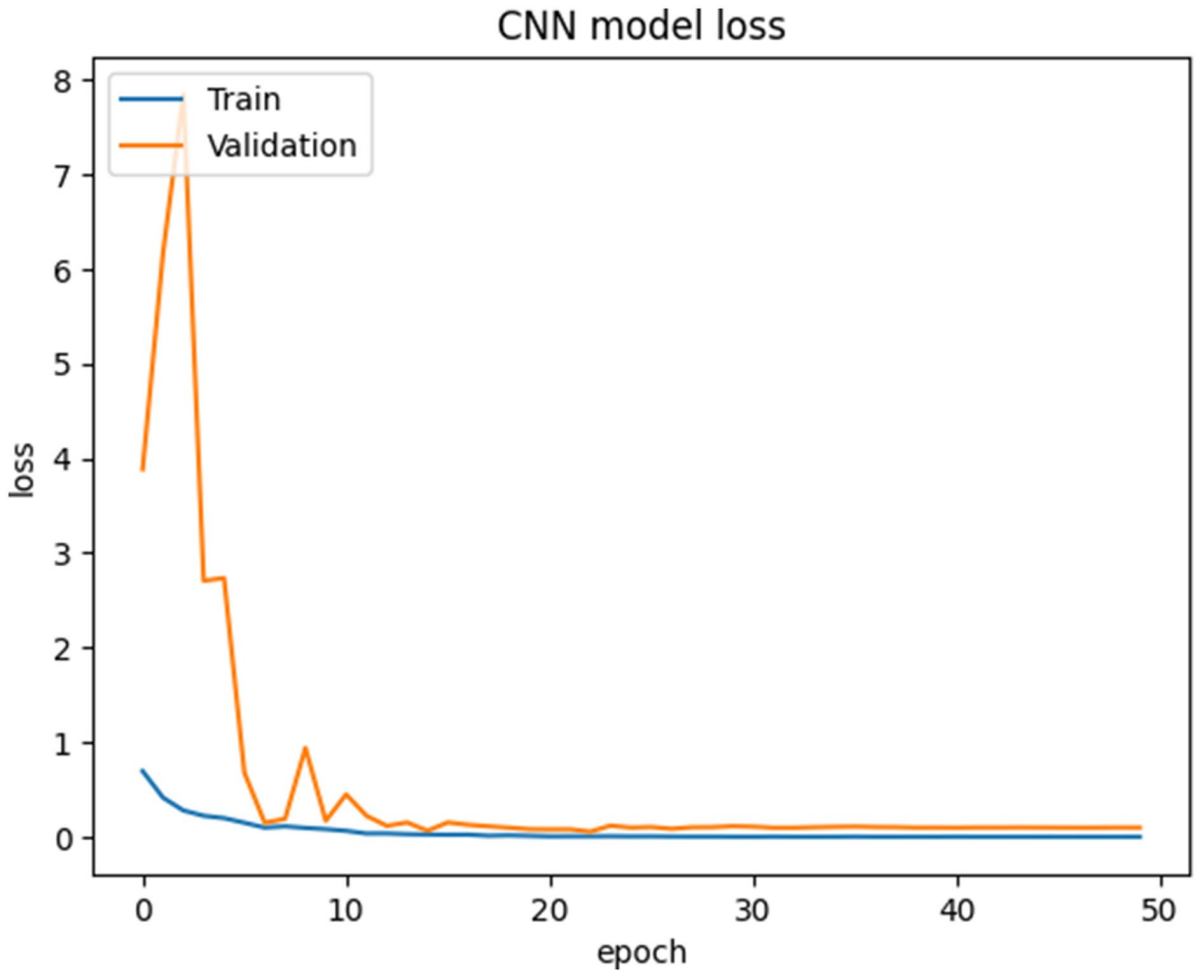
Loss plot for CNN-SEEIB’s performance on potato leaf disease dataset.

**Figure 13:**
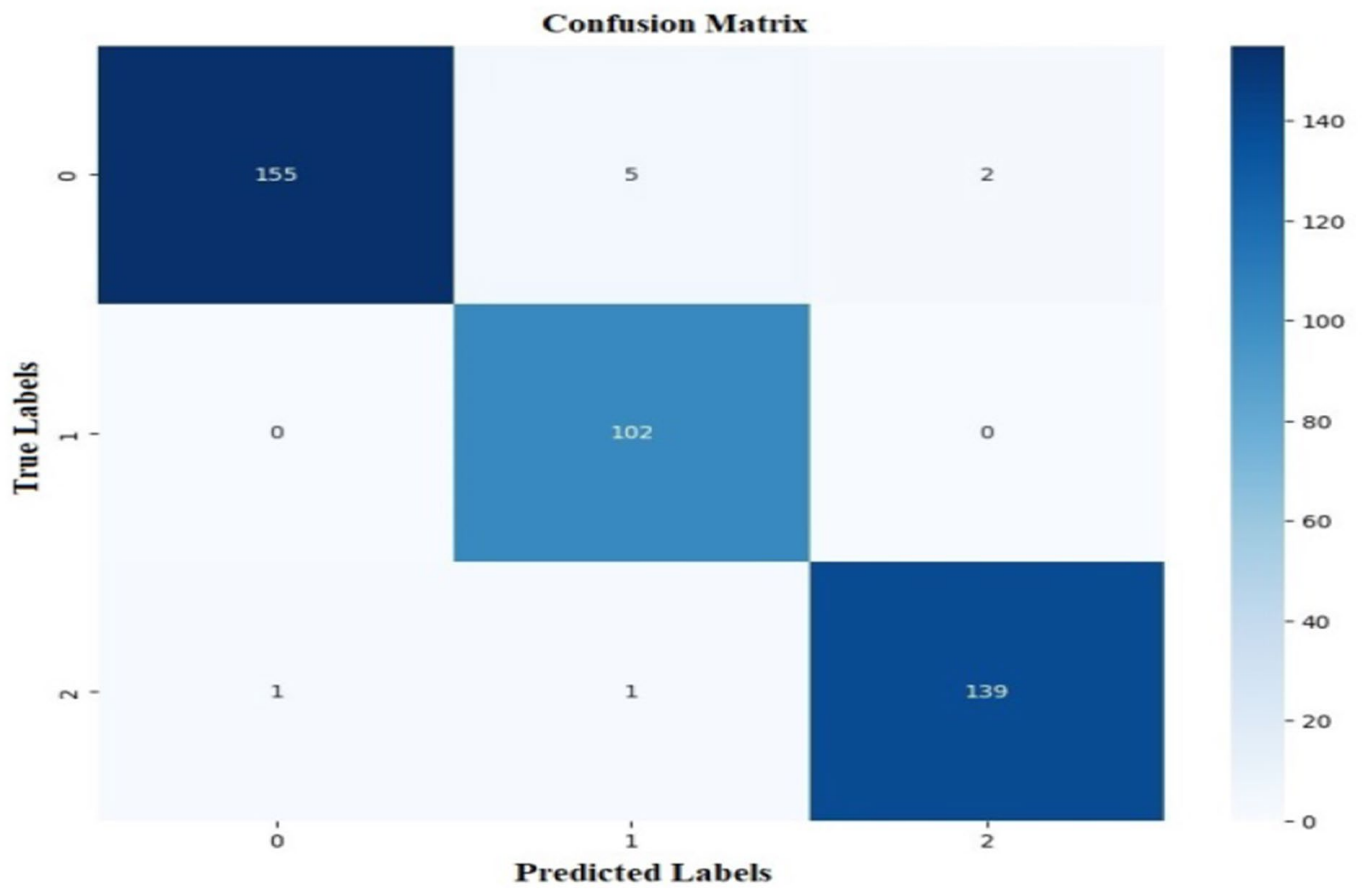
Confusion matrix for CNN-SEEIB’s performance on potato leaf disease dataset.

**Figure 14:**
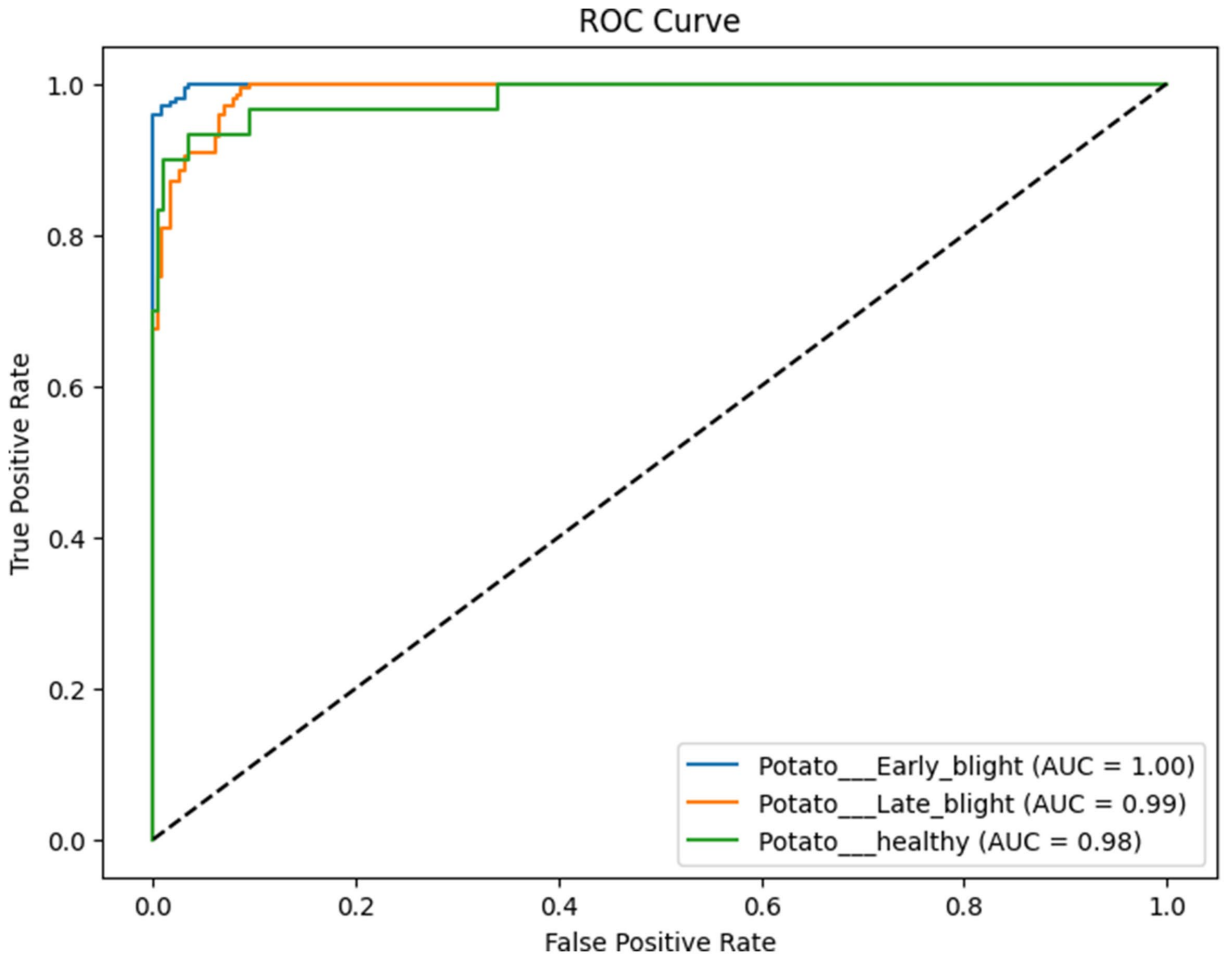
ROC plot for CNN-SEEIB’s performance on potato leaf disease dataset.

**Figure 15:**
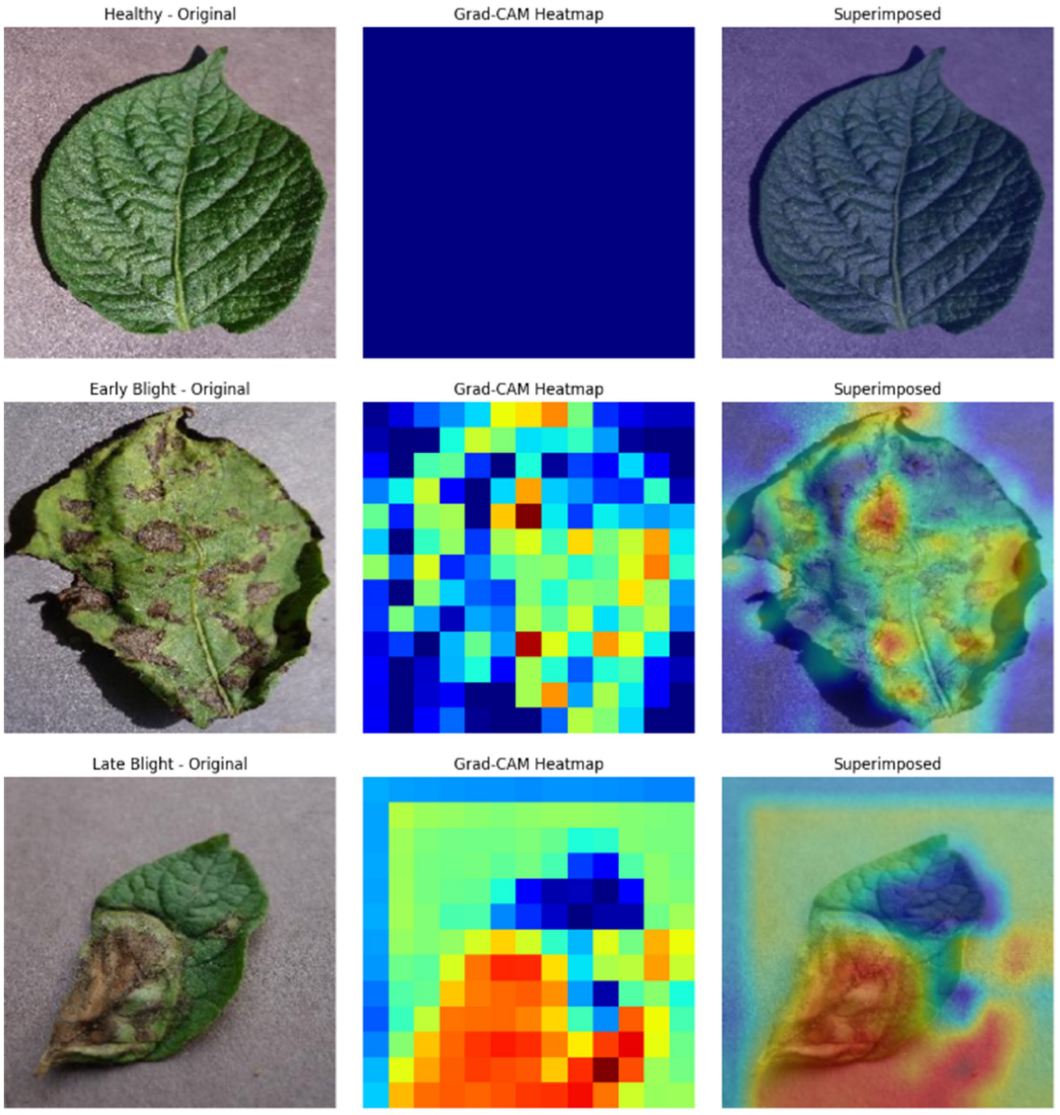
Intermediate feature representation of potato leaf disease.

The proposed CNN-SEEIB was used on this dataset to classify three categories: Early Blight, Healthy, and Late Blight. It achieved an accuracy of 97.77%, precision of 0.9746, recall of 0.9808 and an F1 score of 0.9773. Figures 11, 12 shows the accuracy and loss plot and Figure 13 shows the confusion matrix obtained for the potato leaf disease detection. The confusion matrix shows that Healthy leaves (Class 0) are most often misclassified, with 5 instances as Early Blight (Class 1) and 2 as Late Blight (Class 2), likely due to subtle early-stage symptoms resembling healthy leaves, while Early Blight is correctly classified, indicating its discrete visual markers like well-defined spots that are easily detected. Late Blight (Class 2) has minor errors, with 1 misclassified as Healthy and 1 as Early Blight, possibly due to overlapping features like lesion patterns or discoloration. These misclassifications show the CNN-SEEIB model has to be improved with diseases sharing visual traits, such as spot shapes or color changes. Enhancing the model’s ability to identify fine-grained differences, like texture or spot distribution, an improve the performance on the potato leaf disease dataset. The performance of the proposed CNN-SEEIB model on a dataset distinct from PlantVillage shows its potential for generalization across diverse datasets and real-world scenarios. Figure 14 details ROC Plot for CNN-SEEIB’s performance on potato leaf disease dataset. The inner layers of the proposed CNN-SEEIB model generate visual feature representations, as shown in Figure 15, using Grad-CAM. These Grad-CAM-based visualizations provide a detailed understanding of the discriminative regions the model focuses on while analyzing the potato leaf disease dataset. During training, each layer produces feature maps that capture specific patterns related to disease symptoms. This visualization technique is used in highlighting which regions of the potato leaf images influence the model’s predictions, showing critical insights into the model’s decision-making process and interpretability.

## 5 Conclusion

This paper presents a custom CNN-based model, the CNN-SEEIB that integrates the benefits of accurate and lightweight architectures to automatically classify various plant leaf diseases from images. Initially, a custom backbone CNN architecture was designed, featuring a reduced number of parameters. With this CNN acting as the backbone, attention-based optimizations are incorporated using squeeze and excitation resulting in a unique model with superior classification metrics without significantly increasing parameter count. The experimentation on the PlantVillage dataset shows that the proposed CNN-SEEIB model attained a classification accuracy of 99.79%, precision of 0.9970, recall of 0.9972, and an F1 score of 0.9971. Real-time inferencing revealed an inference time per image of 64 ms. Compared to several transfer learning models, the proposed model outperforms all others in accuracy, precision, recall, and F1-score. With the squeeze-and-excitation model, CNN-SEEIB’s inference time is almost equal to that of MobileNetV2. Also, CNN-SEEIB has the smallest model size and lowest parameter count, while the execution is faster than most pre-trained models except MobileNetV2. To further assess its robustness and generalizability, the model was also evaluated on the potato leaf disease dataset, achieving an accuracy of 97.77%, precision of 0.9746, recall of 0.9808, and F1-score of 0.9773. These results confirm that CNN-SEEIB performs effectively across different datasets and real-world conditions.

Recommendations for further research are as follows:

- Utilizing additional regularization techniques such as bagging and boosting ensemble methods to enhance the generalization capability of the model.
- Extending the classification model to incorporate disease detection and identification functionalities by implementing object detection and segmentation techniques to identify infected regions.
- Augmenting the existing dataset using GANs, NST, and BIM techniques, or combining multiple datasets to increase the number of samples, for reducing the bias and improving model robustness.
- Expanding the number of classes to include a wider range of diseases affecting both leaves, stems and roots, and employing multi-label classification approaches to identify multiple diseases simultaneously.
- Combining other ML techniques for classification, and semi-supervised learning methods to leverage unlabelled data, either imported or self-collected, for model enhancement.
- Exploring various attention mechanisms like self-attention, multi-head attention, soft and hard attention and cross-attention can help recognizing the approach to improve leaf disease detection performance by concentrating on related features and precise patterns.
- Training the proposed model across various other datasets (say, medical images) can help to evaluate its effectiveness and robustness.

## Data Availability

Publicly available datasets were analyzed in this study. This data can be found here: https://www.kaggle.com/datasets/mohitsingh1804/plantvillage.
